# Genome-Wide Identification and In Silico Expression Analysis of *CCO* Gene Family in *Citrus clementina* (Citrus) in Response to Abiotic Stress

**DOI:** 10.3390/plants14020249

**Published:** 2025-01-17

**Authors:** Sadaf Sarwar, Adnan Sami, Muhammad Zeshan Haider, Layba Tasawar, Jannat Akram, Arsalan Ahmad, Muhammad Shafiq, Haitham E. M. Zaki, Gabrijel Ondrasek, Muhammad Shafiq Shahid

**Affiliations:** 1 Department of Horticulture, Faculty of Agricultural Sciences, University of the Punjab, Lahore P.O. Box 54590, Pakistan; 2 Department of Plant Breeding and Genetics, Faculty of Agricultural Sciences, University of the Punjab, Lahore P.O. Box 54590, Pakistan; 3 Department of Entomology, Faculty of Agricultural Sciences, University of the Punjab, Lahore P.O. Box 54590, Pakistan; 4 Horticulture Department, Faculty of Agriculture, Minia University, Minya 61517, Egypt; 5 Applied Biotechnology Department, University of Technology and Applied Sciences-Sur, Sur 411, Oman; 6 Faculty of Agriculture, University of Zagreb, Svetošimunska Cesta 25, 10000 Zagreb, Croatia; 7 Department of Plant Sciences, College of Agricultural and Marine Sciences, Sultan Qaboos University, Muscat 123, Oman

**Keywords:** carotenoid cleavage oxygenase (CCO), phytohormones, gene expression, stress response, genome-wide analysis

## Abstract

The *Citrus clementina* (citrus) plant produces various phytohormones due to the significant involvement of the carotenoid cleavage oxygenase (*CCO*) gene family in its growth and development. *CCO* genes can be divided into two main categories: *NCED* (9-cis-epoxy carotenoid dioxygenase), responsible for abscisic acid (ABA) production, and *CCD* (carotenoid cleavage dioxygenase), involved in pigment and strigolactone formation. To better understand the roles and positions of *CcCCO* gene members in relation to these hormones, researchers analyzed the clementine genome. To identify their structural features, they employed phylogenetic analysis, protein interactions, localization, structure, miRNA targets, evolutionary analysis, and transcriptome studies. The study revealed the presence of 15 *CcCCO* genes, including 11 *NCED* and 4 *CCD* genes, scattered across various chromosomes, with the majority located in chloroplasts. Promoter sequencing analysis indicated the presence of different cis-regulatory elements that likely interacted with phytohormones, such as auxin and abscisic acid among others. Notably, two genes, *CcNCED1* and *CcNCED3*, were significantly expressed among the *CCO* genes, and these were found to be expressed during stress and played a crucial role in enabling optimal plant development. Furthermore, a comprehensive genome-wide comparison of *CCO* genes in *C. Clementine* and *Arabidopsis thaliana* models was conducted to understand their functional characteristics. This research provides a solid foundation for further exploration of the unique attributes of the *C. clementina* plant, contributing to a deeper understanding of its growth and development processes.

## 1. Introduction

Carotenoids signify the class of naturally existing pigments which are prevalent in a broad variety of animals, plants, and microbial organisms [1]. Carotenoids are a group of C40 isoprenoid pigments that are essential for photosynthesis and photoprotection [2]. More than 700 distinctive carotenoids have been observed and characterized since their discovery in the 19th century [3]. The CCO genes mold into an ancient family. The maize (Zea mays) ABA-deficient viviparous mutant, vp14, was the first gene expressing a CCO [4]. Carotenoid cleavage oxygenases (CCOs), as non-heme iron oxygenases, play a crucial role in the oxidative modification of carotenoids, leading to the production of apocarotenoids. These apocarotenoids serve various functions in plants, including acting as phytohormones, pigments, and protective compounds [5]. Plant CCOs can be further split into the NCED (9-cis-epoxy carotenoid dioxygenase) and carotenoid cleavage dioxygenase (CCD) subfamilies based on whether the substrates are epoxidized [6]. However, the RPE65 (retinoid isomer hydrolase RPE65) domain of the NCED and CCD genes is similar as well [7].

The *CCD* gene family in *Arabidopsis thaliana* includes nine members, containing five *NCED* genes (*AtNCED2*, *AtNCED3*, *AtNCED5*, *AtNCED6*, and *AtNCED9*) and four *CCD* genes (*AtCCD1*, *AtCCD4*, *AtCCD7*, and *AtCCD8*) [8]. Recently, the plant species *Vitis vinifera* (grape), *Malus domestica* (apple), and *Solanum lycopersicum* (tomato) were revealed to have the *CCD*-like subtype of *CCO* genes [9,10]. It was discovered that the first *CCD1* enzymes studied (*AtCCD1*) conducted a twofold cleavage of carotenoids, e.g., β-ionone, and one C_14_-dialdehyde, which play an important role in the formation of the flavor and scent of horticultural plants [11]. Natural variation in the *CCD4* gene promoter is a primary genetic predictor of natural variation in C30 apocarotenoids, which cause citrus peel to be red [12]. The *CCD7* and *CCD8* enzymes are involved in the synthesis of strigolactone, which might be able to regulate shoot branching and reproductive development and also affect how plants react to salt and drought stress [13]. Different crop studies of rice (*Oryza sativa*) and maize (*Z. mays*) highlight the variety of roles and activities that *CCD* enzymes play in these plants and their participation in a range of biological processes, such as fruit ripening, hormone production, and plant growth [14,15].

The process mediated by *NCED* is a rate-limiting stage in ABA production, which impacts plant tolerance to various abiotic stressors [16]. Each of the three key components of developmentally mediated ABA production in seeds, *AtNCED5*, *AtNCED6*, and *AtNCED9*, maintains their maturation and dormancy at the embryo stage [8]. In *A. thaliana* roots, *AtNCED2* and *AtNCED3* genes are prevalent, and the proteins they encode contribute to the synthesis of abscisic acid, which controls lateral roots [8]. The significance of *NCED* genes and ABA production in agricultural plants has previously been highlighted by research on tomato (*S. lycopersicum*) and *A. thaliana*, notably in response to water stress and other environmental difficulties [17]. Both enhancing crop resilience and creating tactics for greater stress tolerance in agriculture can benefit from an understanding of the regulation and function of *NCED* genes. The natural levels of ABA in tomato plants are reduced by specifically hindering the function of the *NCED* gene. This drop in ABA level delays the fruit ripening process [18]. A massive 46% decrease in ABA levels appears in strawberry fruits when the *FaNCED1* gene is silenced using the Tobacco Rattle virus [19].

The clementine is a seedless citrus fruit in the *Rutaceae* family, resulting from a cross between mandarin orange and sweet orange. It is renowned for its small size, delicious flavor, and great nutritional content. The commercially grown clementine is an essential component of international agricultural markets and commerce. The genome of the sweet orange, including the clementine variant, was recently sequenced and examined by a global team of academics. This landmark research provided a comprehensive understanding of the genetic makeup of *C. clementina* and laid the foundation for subsequent genomic studies on citrus species. The genome size of *C. clementina* is estimated to be approximately 367 million base pairs (Mb) or 367,000,000 base pairs. The complete genomic sequence of *Citrus clementina* (commonly known as *C. clementine*) was published in 2011 [20].

In citrus plants, the *CCO* family is essential for cellular respiration and energy production. It is found in the mitochondria, where it catalyzes the reduction of molecular oxygen to water while transferring electrons. In the context of gene expression studies, the genes *CcNCED2* and *CcNCED4* demonstrate an increase in expression levels, indicating upregulation. This process generates the energy needed for various metabolic activities within the citrus cells. In our study four *CCD* and eleven *NCEDs* were discovered; these findings open the door for the future. Continued research on *C. Clementina’s* genetic makeup, including genomics and transcriptomics, may unveil new insights into its unique characteristics, leading to the development of novel cultivars and improved cultivation practices to meet evolving consumer demands.

## 2. Results

### 2.1. Identification of CCO Genes in Citrus Clementine

**Figure 1:**
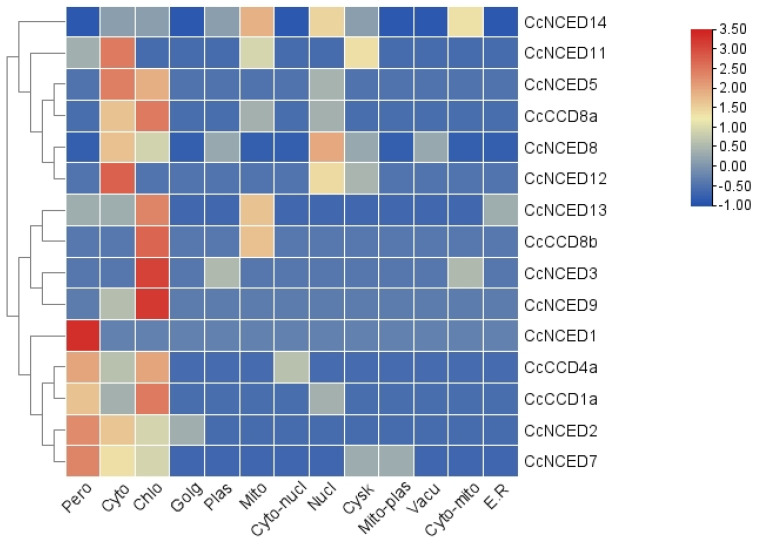
Heat map illustrating the subcellular localization of all 15 *CcCCO* genes to the nucleus, cytoplasm, chloroplast, Golgi apparatus, mitochondria, plasmid, and peroxisomes of the plant cell. The blue color indicates the lowest functional activity or expression of the relevant gene in the specified region, and the red color denotes the highest functional significance of the relevant gene in the indicated region.

There are actually fifteen *CcCCO* genes reported in the *C. clementina* genome (shown in Table 1), of which eleven are *NCED* and four are *CCD*. With *CcNCED12* being the smallest and *CcNCED9* being the longest protein, the *CcCCO* genes that code for it range in size from 410 to 609 amino acids (AA) and from 45,860.27 to 69,176.61 Da molecular weight (Mw). The identified proteins have pI values ranging from 5.79 to 8.53. These 15 *CcCCO* genes were subcellular localized, and the bulk of the genes were found in the cytoplasm, peroxisomes, and chloroplast (Figure 1). Some were found in the nucleus and mitochondria. The last of them were also found in plasmids, extracellular structures, vacuoles, and E.R, etc.

### 2.2. Phylogenetic Analysis

**Figure 2:**
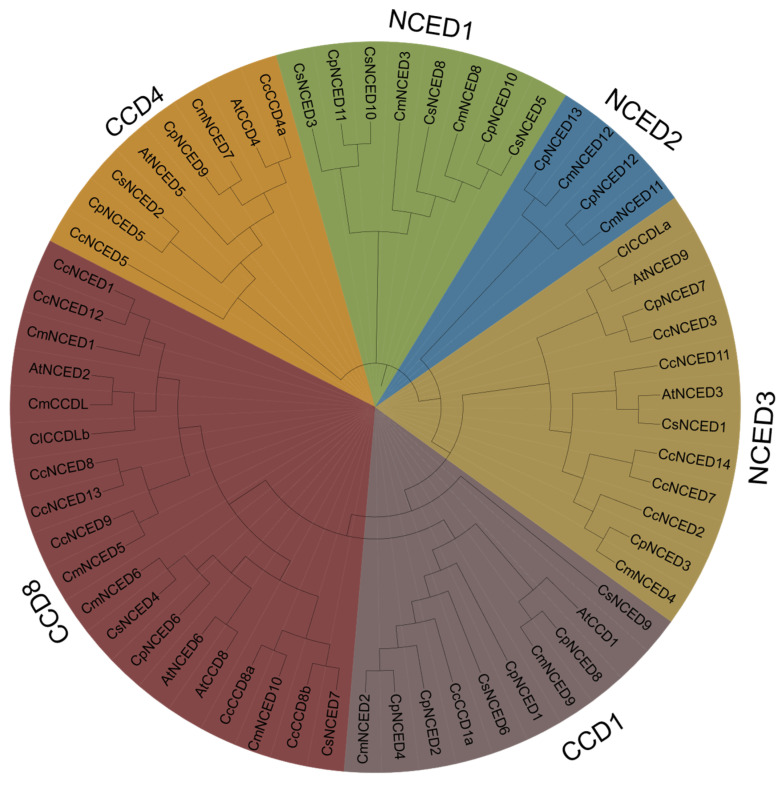
A phylogenetic tree restoration method, which is often employed used in research on *A. thalianan*, was utilized to determine the phylogenetic connections of 61 *CCO* genes from five different families, including *C. clementine*, *C. sativus*, *A. thaliana*, *C. maxima*, and *C. pepo*. Based on their associated evolutionary features and traits, the five crop species are split among the 61 *CCO* genes in the figure.

The reference sequences of 15 CcCCO genes from *C. clementina*, 10 genes from *C. sativus*, 8 genes from *A. thalianan*, 12 genes from *C. maxima*, and 13 genes from *C. pepo* were used to construct a phylogenetic tree. All 61 CCO genes were utilized for the tree. The phylogenic investigation revealed that the *CcCCO* genes were made up of three *NCED* genes and four *CCD* genes. The phylogenetic tree was categorized based on the presence of *A. thaliana* genes in each clade. According to the analysis, the CcCCO genes can be classified into two main groups, CCD and NCED, which are further subdivided into six subgroups: CCD1, CCD4, CCD8, NCED1, NCED2, and NCED3. Among these, CCD8 is the largest subgroup with 19 genes, followed by NCED3 with 12 genes. CCD1 ranks third with 10 genes, while NCED1 and CCD4 contain 8 and 10 genes, respectively. The smallest group is NCED2, which consists of only four genes (Figure 2). A clementine gene was not found in the CCDL subgroup, which is noteworthy. The study revealed that citrus clementine harbors two genes in the CCD8 clade and five genes in the NCED3 clade. In contrast, NCED1 and NCED2 lack genes, while CCD1 possesses only one gene. Interestingly, *CCDL* genes form a distinct clade in another crop. Their significance remains uncertain due to their absence in *A. thalianan*. The CCD4 clade contains two Cp genes and one gene each from Cs and Cm. Within the NCED1 clade, there are four Cs genes, accompanied by two genes from Cp and Cm. The CCD8 clade comprises five, one, and two genes from Cm, Cp, and Cs. Finally, NCED2 encompasses two genes, originating from Cp and Cm.

### 2.3. Gene Structure, Motif, and Domain Analysis

**Figure 3:**
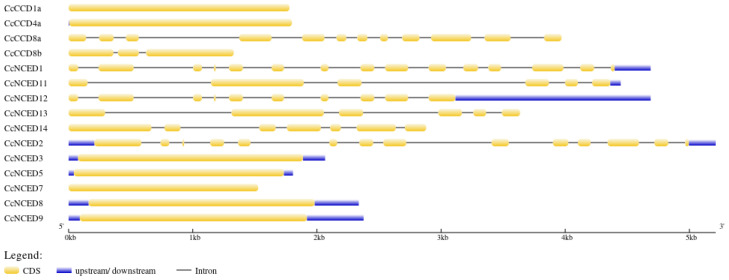
The intron–exon structure is phylogenetically represented, and it shows that *CCD* genes contain fewer coding sequences than *NCED* genes. The number of introns and exons in several *NCED* and *CCD* genes has remained constant.

In accordance with the *CcCCO* gene structure suggested by the intron–exon analysis, seven of the fifteen genes—*CcCCD1a*, *CcCCD4a*, *CcNCED3*, *CcNCED5*, *CcNCED7*, *CcNCED8*, and *CcNCED9* have one exon and no intron. Six exons and five introns exist in the two genes *CcNCED11* and *CcNCED13*. Other genes include *CcNCED1*, which has 15 exons and 14 introns, *CcNCED12* which has 10 exons and 9 introns, *CcCCD8b* which has 3 exons and 2 introns, *CcNCED14* which has 7 exons and 6 introns, and *CcNCED2* which has 14 exons and 13 introns (Figure 3). This research demonstrates that, in contrast to other genes, those genes with the same number of introns and exons share common jobs and ancestry.

**Figure 4:**
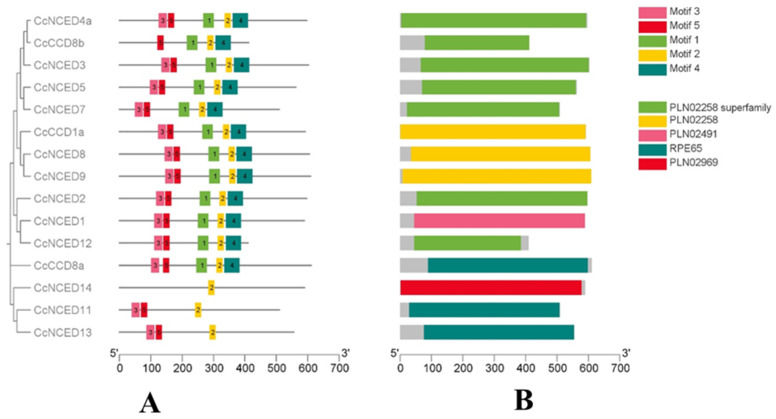
The distribution of 20 motifs along the 15 members of the citrus clementine *CCO* protein family. Some members of the *NCED* and *CCD* proteins have a conserved motif shown in (**A**), while the conserved domain in each of the CCO genes is depicted in (**B**).

Fifteen *CcCCO* genes were subjected to motif analysis, revealing the presence of 20 different motifs. Interestingly, one pattern was found to be conserved in all 15 genes, indicating that it is vital to their operation (Figure 4). Motif 2 is conserved in all identified CCO genes while motif 5 is present in 14 *CcCCO* genes out of 15. In addition to their respective domain analyses, the RPE65 domain is the prominent domain present in CCO genes and has subdomains such as PLN02258, PLN02969, and PLN02491 that are present in *CcCCO* genes (Figure 4).

### 2.4. Evaluation of Duplication Event of C. clementine

**Figure 5:**
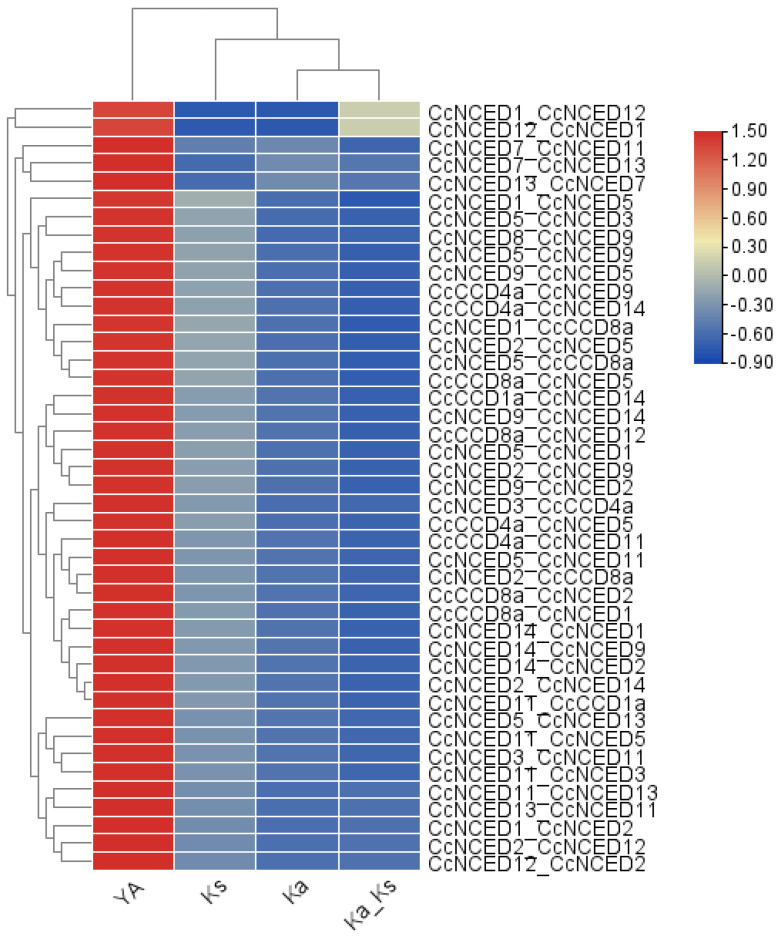
The expression ka/ks represents the ratio of mutations involving synonymous substitutions (ks) to mutations involving nonsynonymous substitutions (ka). The gene duplication over selection and evolutionary pressure to paralogous pairings of *C. clementina CCO* genes were calculated based on ks and ka values.

Through the use of a relatively simple ka/ks calculator, TB tools determined the values of ks, ka, and the ka/ks ratio of the *CcCCO* genes. The ratio of synonymous to nonsynonymous changes was expressed as ka/ks, where ks represents the number of synonymous substitutions per synonymous site and ka represents the number of nonsynonymous substitutions per nonsynonymous site. This ratio ranged from 1.98 in the *CcNCED7*/*CcNCED13* pair to 0.08 in the *CcNCED8*/*CcNCED9* pair. When all of the genes’ ka/ks values are less than 1, all of the genes are subject to replacement, which preserves the functional integrity of the gene by eradicating undesirable modifications. All paralogous pairs in citrus clementine had a Ka/Ks ratio greater than 0.05 which suggested the probability of significant functional divergence in the duplication process due to purifying selection (Figure 5).

**Figure 6:**
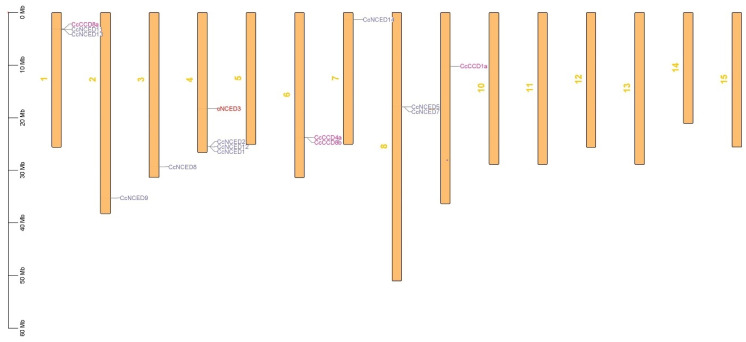
The *C. clementina* genome’s chromosomal mapping of the *CCO* genes shows the existence of paralogous copies with plausible locations.

Based on their chromosomal positioning, the chromosomal localization study suggested that *CcCCO* genes were meant to be divided over several chromosomes. On the first spot, *CcCCD8a*, *CcNCED13*, and *CcNCED11* were present. *CcNCED3*, *CcNCED2*, *CcNCED12*, and *CcNCED1* were present on the fourth position. On position six, *CcCCD4a* and *CcCCD8b* were seen. The locations of *CcNCED5* and *CcNCED7* were eight and nine, respectively. On two, three, seven, and ten there were instances of *CcNCED9*, *CcNCED8*, *CcNCED14*, and *CcCCD1a.* The clustering of *CcCCO* genes on chromosomes 1, 4, 5, and 8 may reflect functional or regulatory coherence, as functional clusters are known to enhance co-expression and coordinated regulation in plants and other organisms (Figure 6). This phenomenon could be the result of selection pressure, gene duplication events, and chromosomal rearrangements that facilitated the retention of essential functions in the *C. clementina* genome. The clustering may also indicate a role in metabolic pathways or stress responses, consistent with findings in other organisms [21].

**Figure 7:**
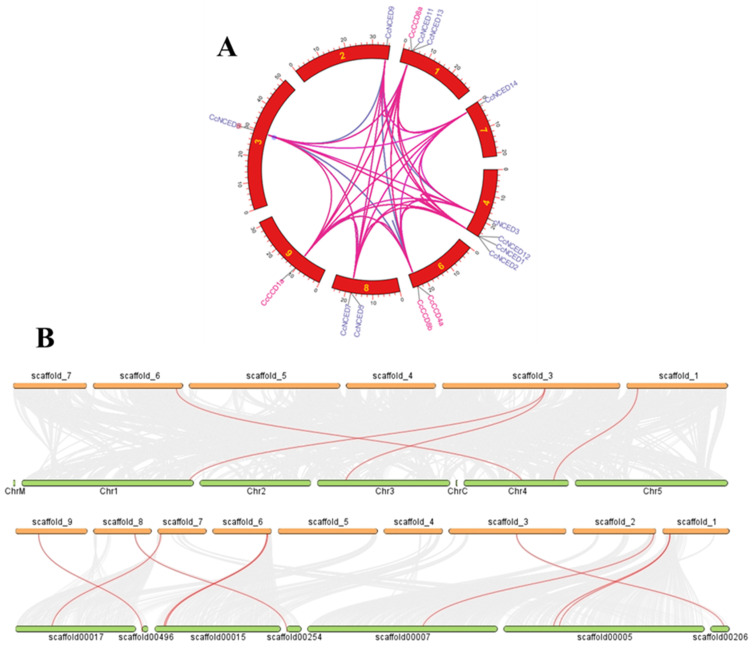
Genes with structurally similar sequences share conserved areas, according to an in-depth study of genome-wide synteny in clementine *CCO* genes (**A**)**.** Dual synteny examinations were carried out to assess the degree of structural similarity and the distribution of gene copies between the genomes of *C. clementina*, *A. thalianan*, and *C. clementina*–*Citrus sinensis* (**B**).

Synteny offers a structural foundation for understanding the preservation of similar genes and their orderly organization across genomes of the same species. It involves identifying conserved regions between two sets of chromosomes that are being compared. Dual synteny is the term used to describe the conserved pairing of genes on chromosomes in different animals. Two genetic sites may become separated through evolutionary changes to the genome, such as chromosomal translocations, which can separate two genetic positions. This technique gives a visual representation of the degree of similarity through genomic regions using pink and blue shades. Gene duplication is a result of an array of dynamic mechanisms and genomic rearrangements that allow the genes to remain stable secure, adapt to their environment, and gain new properties over time (Figure 7A). In the genomes of these plant species, our research revealed instances of slight structural and gene duplication sharing (Figure 7B).

### 2.5. Analysis of CcCCO Cis-Regulatory Elements

**Figure 8:**
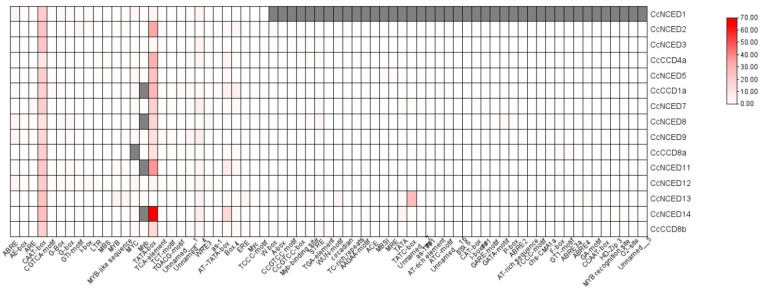
The visual depiction of the *CCO* gene’s cis-regulatory analysis along with the strength of each function. The red (highest) to the white (lowest) intensity is used to describe the biochemical and physiological activities happening in plants.

The spatio-temporal transcriptomic expression of genes is influenced by the presence and arrangement of numerous cis-regulatory elements at the binding site of transcription factors on the promoter region. Therefore, research using the PlantCare database was undertaken to assess the possible roles of *CcCCO* genes. Light-responsive, endosperm-specific, hormone-specific, meristem-specific, metabolism-related, stress and defense-related cis-regulatory elements made up of clementine *CCO* genes were studied. The *CCO* gene family in *C. clementina* contained 69 cis-regulatory elements that are responsive to light (Figure 8).

The ABA reaction is regulated by the AAGAA motif element, a basal promoter element with the ATTATA-box structure. BOX 4, and G-Box engaged in reactivity to light. The CAAT box element controls meristem expression. Meanwhile, the MeJA responsiveness of the CGTCA motif element is also important. The MYB is involved in cell proliferation and the cell cycle. Myb, AT-Rich Element, MRE, ERE, CGTCA-motif, and STRE all exhibit wound responsiveness. The WUN motif and W-box trigger a protective reaction. Defense and stress reactivity is influenced by TC-rich proteins, and GATA-motif, LTR, LAMP-element, CGTCA-motif, MBS, and TCT-motif elements each serve a specialized purpose. Both AE-box and ARE are crucial for triggering aerobic induction. All other elements, including ACE, TGA element, circadian, MYB-like sequence, Myb-binding site, GTI-motif, Unnamed_2, Unnamed_16, I-box, P-box, and GARE-motif, are engaged in various roles.

### 2.6. Analysis of Protein–Protein Interaction Network

**Figure 9:**
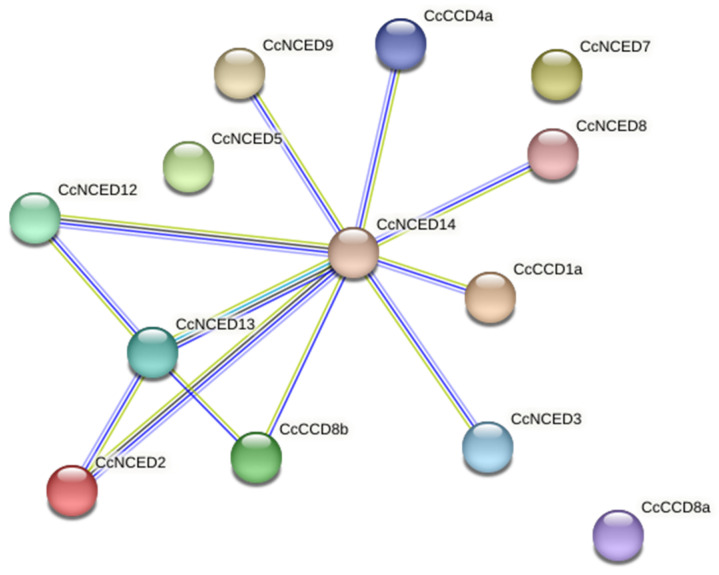
Protein–protein interaction network of CcNCED14, CcCCD8a, CcNCED11, and CcNCED1.

The *CcNCED14* gene has associations with all the other genes, according to protein–protein interaction, although *CcCCD8a*, *CcNCED11*, and *CcNCED1* also share distinct bonds with one another. *CcNCED14* interacts with an array of different proteins in the dataset. *CcNCED14* may engage in a variety of connections and participate in several cellular processes. *CcCCD8a*, *CcNCED11*, and *CcNCED1* form a sub-network in the larger protein–protein interactions network that uniquely interacts with one another. These proteins may take part in a specific biological process or have a coordinated functional relationship (Figure 9). *CcNCED14* proteins demonstrated interactions with the following different proteins, *CcNCED7*, *CcNCED8*, *CcCCD4a*, *CcCCD1a*, *CcNCED9*, *CcNCED5*, *CcNCED3*, *CcNCED12*, *CcNCED13*, *CcCCD8b*, and *CcNCED2*, except for *CcCCD8a*, *CcNCED11*, and *CcNCED1*. The STRING database delivered information on several functional enrichments, including molecular activities, biological processes, and KEGG pathways, that were present among the protein interactions for *CcNCED7*, *CcNCED8*, *CcCCD4a*, *CcCCD1a*, *CcNCED9*, *CcNCED5*, *CcNCED3*, *CcNCED12*, *CcNCED14*, *CcNCED13*, *CcCCD8b*, and *CcNCED2*. Gene ontology presented all *CcCCO* proteins, biological, cellular, and molecular activities. All genes were significantly enriched in biological activity, including the catabolic processes for beta-carotene, carotene, and abscisic acid, as well as the biosynthetic processes for strigolactone. This was verified through GO functional annotation and enrichment analysis.

### 2.7. MicroRNA Target Site Analysis

Six distinct genes, *CcCCD1a*, *CcCCD8a*, *CcNCED5*, *CcNCED7*, *CcNCED8*, and *CcNCED9*, were targeted by a total of fifteen reported miRNAs. *CcNCED9* was the gene with the highest number of miRNA targets, whereas, *CcCCD1a*, *CcCCD8a*, and *CcNCED8* had the lowest numbers.

The upregulation of *CcNCED8*, *CcNCED7*, *CcNCED5*, and *CcNCED9* under stress scenarios such as drought, heat, or salinity results in an increase in ABA production and contributes to the fruit’s expansion, stress responses, and general physiology. On the contrary, *CcCCD1a’s* main task includes the production of volatile compounds and their impact on fruit aroma, ripening, and fruit development. The generation of certain apocarotenoids, which lead to the fruit’s distinctive qualities, is influenced by *CcCCD8a*.

### 2.8. Gene Expression

The relative expression levels of *CcNCED* and *CcCCD* genes in *C. clementina* (CcTJ) and *Citrus japonica* (JG9) were analyzed under abiotic stress conditions. The results revealed significant differences in gene expression between the two species for several genes. Notably, *CcNCED1* showed a higher expression in *C. japonica* (184.78) compared with *C. clementina* (123.22), with a log2 fold change of 0.58 and a *p*-value of 0.001, indicating upregulation. Conversely, *CcNCED2* and *CcNCED4* exhibited lower expression levels in *C. japonica* than in *C. clementina*, with log2 fold changes of −1.12 and −1.23, respectively, and significant *p*-values (0.00028 and 0.00025). Similarly, *CcNCED8* was upregulated in *C. japonica* with a log2 fold change of 1.03 (*p* = 0.00049).

**Figure 10:**
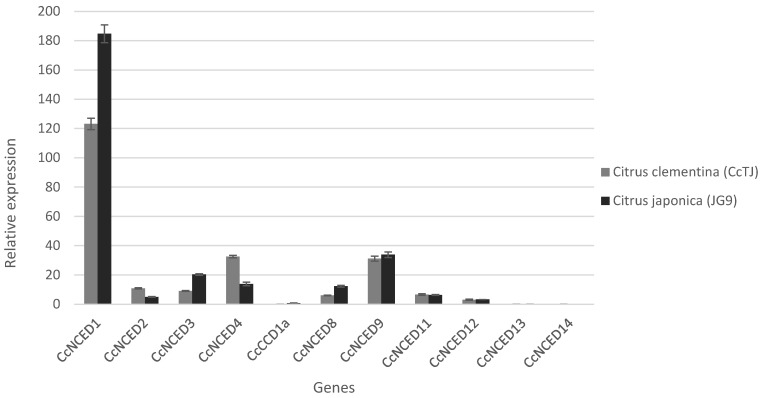
Graph displaying gene expression. A vertical bar graph with time intervals on the x-axis and expression levels on the y-axis depicts the expression of *CCO* genes. Each color represents a specific gene as shown in the legends, the error bar is represented by a black colored line (plus cap).

Interestingly, *CcCCD1a* showed a substantial increase in *C. japonica* (0.7) compared with *C. clementina* (0.023), with a log2 fold change of 4.91 (*p* = 0.027). However, *CcNCED9*, *CcNCED11*, *CcNCED12*, *CcNCED13*, and *CcNCED14* did not display significant changes between the two species (*p* > 0.05). Overall, the results highlight distinct expression patterns of the *CcNCED* and *CcCCD* genes in *C. clementina* compared with *C. japonica*, indicating species-specific responses to abiotic stress, with *C. clementina* showing more stable expression in certain genes. These findings emphasize the functional divergence of these genes in the stress response of citrus species (Figure 10).

## 3. Materials and Methods

### 3.1. Sequence Retrieval

To determine the amino acid sequence linked to the *CCO* domain, a search was undertaken in the NCBI (https://www.ncbi.nlm.nih.gov) database. In the subsequent BLAST-P (Protein-basic local alignment search tool program) analysis, the *C. clementina* genome database (cucurbitgenomics.org/blast, accessed on 2 July 2024) was utilized as the target and these sequences, specifically the RPE65 domain, were employed [9]. Fifteen sequences from the *C. clementina* database were identified as a consequence of this research. The NCBI CCD (Conserved Domain Database, http://www.ncbi.nlm.nih.gov/Structure/cdd/wrpsb.cgi, accessed on 2 July 2024) with preset parameters was used to verify the precision of these sequences. Proteins that lacked the conserved domain underwent comprehensive analysis before being eliminated from further evaluation.

### 3.2. Determination of Physiochemical Properties and Subcellular Localization of CCO Genes

To construct predictions regarding the *CCO* proteins, the ProtParam online tool (https://web.expasy.org/protparam/, accessed on 14 July 2024) was employed [20]. The protein length, molecular weight, and isoelectric point (pI) were all quantified using this approach. The *C. clementina* genome database was used to acquire the names of the genes, their positions on the chromosomes, and the protein sequence of the *CCO* proteins. To determine the subcellular location of the *CCO* genes, the WoLF PSORT program (https://wolfpsort.hgc.jp/, accessed on 15 July 2024) was also used [20].

### 3.3. Gene Structure, Cis-Regulatory Analysis and Motif Analysis

The intron–exon structure of the *CCO* genes was displayed using the Gene Structure Display Server (GSDS) v2.0 (http://gsds.cbi.pku.edu.cn/, accessed on 4 August 2024). The PlantCare database (http://bioinformatics.psb.ugent.be/webtools/plantcare/html, accessed on 10 August 2024) was used to investigate the cis-regulatory elements linked to these genes. To find motifs, the MEME suit program (http://meme.nbcr.net/, accessed on 10 August 2024) was utilized with a maximum value of 25 and TB tools was used to display the found motifs [22].

### 3.4. The Analysis of Phylogenetic

Molecular Evolutionary Genetics (MEGA-11) software was used in the study to carry out a phylogenetic analysis [23]. The MUSCLE approach was first used to align the amino acid sequences of the *CCO* proteins of *Cucumis sativus*, *A. thalia*, *Cucurbita pepo*, *Cucumis lanatus*, *Cucumis mochata*, and *C. clementina*. The neighbor-joining (NJ) approach was used to create a phylogenetic tree from the aligned protein sequence with a bootstrapping value of 1000 replications. iTOL (https://itol.embl.de/upload.cgi, accessed on 15 August 2024) was used to show and visualize the derived phylogenetic tree [23].

### 3.5. Gene Duplication and Synteny Analysis

TB tools was used to figure out the *CCO* gene divergence period using the Ka/Ks ratio. The gene pair was calculated by measuring the Ka/Ks ratio using paralogous genes [24]. Then, using the formula T = Ks/2r, where r stands for the neutral substitution rate, the time of divergence (DT) was determined. MCScanX v1.0 (Multiple Collinearity Scan toolbox) was used with default settings to evaluate gene duplication occurrences. *A. thaliana* and Buttercup Squash were used in the dual synteny study of the two crops. The TB tools circus module was used to build the synteny graph [25].

### 3.6. Gene Ontology Analysis and Protein–Protein Interaction

Gene ontology (GO) term enrichment analysis using GO annotations from the uniprot website (https://www.uniprot.org/, accessed on 19 August 2024) was used to corroborate the *CCO* gene’s roles in *C. clementina*. ShinyGo v0.741 was used to better understand the molecular functions and biological processes connected to these genes. Furthermore, research on protein interactions was conducted using the STRING Database (https://string-db.org/, accessed on 19 August 2024) for protein interaction studies.

### 3.7. Putative miRNA Analysis

The National Center for Biotechnology Information (NCBI) determined the miRNA sequence for the mature *C. clementina* plant. The coding sequence (CDS) of all the *CCO* genes was used to determine the micro-RNA (miRNA) sequences associated with them using the psRNATarget tool, which is available at (https://www.zhaolab.org/psRNATarget/, accessed on 21 August 2024) [26].

### 3.8. Chromosomal Mapping

The clementine genome database was used to derive the start and stop sites of the CCO gene. The chromosomal mapping of the gene was shown using TB tools, making it clear.

### 3.9. Expression Analysis

A range of plant parts, including phloem-rich leaves, pedicles, stalks, and fruits, as well as various biological circumstances, were used to measure the expression levels of all *CCO* genes. High-throughput sequencing data that had been previously gathered were used to undertake the expression study of these *CCO* genes (https://www.ncbi.nlm.nih.gov/geo/query/acc.cgi?acc=GSE107812, accessed on 1 September 2024) [27]. The NCBI Gene Expression Omnibus (GEO) was a helpful tool for learning about the phases of organ development and their reactions to various biotic and abiotic stimuli [24]. And, while preparing this article, the authors used ChatGPT v.4 to enhance language and readability. Following this tool, the authors thoroughly reviewed and edited the content as necessary.

## 4. Discussion

*C. clementina* is a very important agricultural species that is cultivated all over the world [28]. Its current yearly production is predicted to be over 105 million tons; along with its great yield potential and nutritious values, it is seen as a promising crop for easing human hunger and poverty. Although *C. clementina* crops tend to be raised by resource-limited farmers in less developed countries, they are prone to production losses carried by various abiotic and biotic issues [29]. Plants may deal with external environmental issues thanks to transcription factors (TFs), and families including MYB, bHLH, WRKY, NAC, bZIP, and AP2 are important for minimizing yield losses [30]. The clementine TF family is in one of these families that has earned a lot of attention in plant research, especially due to its role in regulating secondary metabolism, pathogen defense, seed development, and responses to abiotic stress in citrus species [31].

The essential hormone ABA, which is vital for plant growth, development, and stress resistance, is produced by the rate-limiting enzyme 9 cis-epoxycarotenoid dioxygenease (NCED) [32]. In the current study, a total of 15 *CCO* genes in clementine were found. These discoveries provide insight into the processes that lead to the creation of new gene families and novel gene functionality, a key factor promoting the emergence of novel genes and enhancing their activities [33]. In particular, the *CcCCO* family is typically seen in various plant species.

*NCED* genes, such as those of the *C. clementina*, have been well studied in dicotyledonous plants such as *A. thalianan* [34]. As such, genome-wide analysis was conducted to identify and characterize the CCO genes in clementine. The study identified a total of 15 CCO genes and revealed variations in the number of exons and introns for each gene, potentially contributing to functional diversity. Additionally, the analysis identified diverse subcellular localizations for the CCO genes, including the nucleus, cytoplasm, and chloroplast. Promoter analysis revealed various cis-regulatory elements associated with plant growth, development, light response, and stress adaptation. These findings suggest that CCO genes play a crucial role in vegetative growth and contribute to mitigating both biotic and abiotic stress conditions [31].

The expression patterns of various genes were investigated under different stress situations with the intent to better understand the potential roles of *CcCCO* genes in the *Rutaceae* family. These findings imply that *CcCCO* genes have an array of roles in plant growth and development and may help plants adapt to a variety of stressors.

Gene duplication is a common evolutionary process where a preexisting gene in an organism’s genome is duplicated. This can occur through various mechanisms such as retro-transposition, whole-genome duplication, or inconsistent crossing over. Gene duplication is crucial for evolution because it allows duplicated genes to acquire new functions, increasing complexity and diversity. This can happen through sub-functionalization and neo-functionalization [35]. Synteny analysis is a valuable method for studying gene duplication events and their impact on evolution. It involves assessing gene arrangement and sequencing across chromosomes or within a genome, helping researchers identify duplications and understand gene family evolution and functional diversity.

Their chromosomal position may be utilized to predict this. This means that genes located on the same chromosome might theoretically be the outcome of segmented duplication [36]. Segmented duplication occurred primarily in the case of the *CcCCO* genes. Gene duplication is the main driver of genome expansion in species [37]. Variations in the gene family members across different eukaryotic species might be because of the process of evolution [38].

The way proteins interact with other proteins reflects a number of crucial plant processes and actions, including physiological aspects, involving physiological, pathological, and developmental processes as well as signal transduction pathways [39]. The STRING database showed that different functional enrichments exist in *CcCCOs* proteins, including molecular functions, biological processes, and the KEGG pathway.

Furthermore, the dual synteny blocks within the clementine’s genome were established using three distinct crops, *A. thalianan* and *C. sinensis*, which were researched individually to clarify their links. Two genes on chromosome 4 and one gene on each of chromosomes 1 and 3 were duplicated in *A. thalianan*. These relationships were visually represented using connecting threads between the corresponding chromosomes. Similarly, the synteny block comparison between *C. clementina* and *C. sinensis* revealed that the two species shared eight duplicated genes originating from different chromosomes, which were also depicted using connecting threads.

MicroRNAs (miRNAs) are important regulatory entities of plants [40]. They have a role in the regulation of almost all biological processes of plants, such as plant growth and development, during biotic and abiotic stress. They are highly conserved and are very specific in function. A putative miRNA study showed the miRNAs which targeted *CcCCO* genes. It was found that seven miRNAs targeted *CcNCED9*, while one miRNA targeted *CcNCED8*, *CcCCD8a*, and *CcCCD1a* each. This family’s main task includes the production of volatile compounds and their impact on fruit aroma, ripening, and fruit development. *CcNCED7* and *CcNCED5* were targeted by two miRNAs, respectively [31].

## 5. Conclusions

In this study, 15 *CcCCO* genes were found in *C. clementina*. The number of introns in *CcCCO* genes varies from one to fourteen, based on structural analysis. The promoter of the *CcCCO* genes contained cis-regulatory elements related to light responsiveness, cell proliferation, aerobic induction, hormone responsiveness, and certain abiotic stress, which suggested that these elements play a role in the abiotic stress suffered by *C. clementina*. The overexpressing specific genes such as *CcNCED1* and *CcNCED3*, along with suppressing downregulated genes, could contribute to developing stress-resistant clementine cultivars, thereby enhancing yield production.

## Figures and Tables

**Table 1:** *NCED* and *CCD* genes in *CCO* gene family information for 15 non-redundant genes discovered in the *C. clementina* genome.

Gene ID	Source Accession No.	Chromosome No.	Chromosome Location		Direction	pI	No. of AA	Mw (Da)	Size	
Name	Phytozome ID	Scaffold	Start	End					Genome	Peptide
**CcNCED1**	Ciclev10031039m.1	4	25506445	25511133	F	6.05	589	66,677.35	4688	590
**CcNCED2**	Ciclev10031014m.1	4	25500600	25505812	F	6.33	597	67,203.01	5212	598
**CcNCED3**	Ciclev10031003m.1	4	18266261	18268327	R	6.87	603	66,452.87	2066	604
**CcCCD4a**	Ciclev10011335m.1	6	23758343	23760142	F	8.53	597	66,347.09	1799	598
**CcNCED5**	Ciclev10028113m.1	8	17933011	17934818	F	8.34	563	63,060.24	1807	564
**CcCCD1a**	Ciclev10006710m.1	9	10202438	10204217	R	7.32	592	65,292.48	1779	593
**CcNCED7**	Ciclev10030384m.1	8	17966529	17968056	F	6.56	508	56,910.31	1527	509
**CcNCED8**	Ciclev10019364m.1	3	29351853	29354190	R	6.37	606	67,013.31	2337	607
**CcNCED9**	Ciclev10014639m.1	2	35235516	35237892	F	6.30	609	67,794.81	2376	610
**CcCCD8a**	Ciclev10010551m.1	1	3113082	3117053	R	5.79	611	69,176.61	3971	612
**CcCCD8b**	Ciclev10013726m.1	6	23780381	23781711	F	7.25	412	45,860.27	1330	412
**CcNCED11**	Ciclev10008050m.1	1	3187977	3192424	F	5.93	510	56,800.61	4447	511
**CcNCED12**	Ciclev10031690m.1	4	25506445	25511133	F	8.27	410	46,734.14		
**CcNCED13**	Ciclev10010609m.1	1	3208798	3212434	F	5.98	556	61,964.53	3636	557
**CcNCED14**	Ciclev10027500m.1	7	1348302	1351182	R	6.07	590	66,508.85	2880	591

Abbreviations: AA: amino acid, Mw: molecular weight, pI: isoelectric point.

## Data Availability

I affirm that all necessary data and permissions have been provided for this study. Any interested researchers can access the required data to support the findings and conclusions of this article. For publicly archived datasets, hyperlinks are provided in this manuscript in appropriate place for convenience.

## Declarations

Rest assured, I have ensured that all data, materials, software applications, and custom code supporting the claims made in this article are in full compliance with field standards. It is important to note that I have taken into account the possibility of individual journal policies regarding research data sharing, considering the norms and expectations of our discipline.
